# Knowledge, attitude, and practice of birth attendants regarding partograph use and its association with birth outcomes: a cross sectional study in Iran

**DOI:** 10.1186/s12884-026-09569-1

**Published:** 2026-07-03

**Authors:** Leila Rastegari, Nahid Javadifar, Nahid Shahbazian, Azam Bagheri, Saeed Ghanbari

**Affiliations:** 1 Midwifery Department, Ahvaz Jundishapur University of Medical Sciences, Ahvaz, Iran; 2 Reproductive Health Promotion Research Center, Ahvaz Jundishapur University of Medical Sciences, Ahvaz, Iran; 3 Fertility, Infertility and Perinatology Research Center, Ahvaz Jundishapur University of Medical Sciences, Ahvaz, Iran; 4 Department of Midwifery and Reproductive Health, Kashan University of Medical Sciences, Kashan, Iran; 5 Department of Biostatistics and Epidemiology, School of Health, Ahvaz Jundishapur University of Medical Sciences, Ahvaz, Iran

**Keywords:** Partograph, Knowledge, Attitude, Practice, Birth, Outcomes

## Abstract

**Background:**

The partograph is a World Health Organization (WHO)-recommended tool for monitoring labor and supporting timely clinical decisions. Despite its importance, its use remains suboptimal in many low- and middle-income settings, with persistent gaps between knowledge and practice. Evidence from Iran across different categories of birth attendants is limited. This study therefore assessed partograph use among midwives, obstetrics residents, and midwifery students, and its association with selected maternal and neonatal outcomes in teaching hospitals in Ahvaz, southwest Iran.

**Methods:**

This cross-sectional study was conducted in 2023 in four teaching hospitals in Ahvaz, southwest Iran. A total of 155 birth attendants completed self-administered knowledge and attitude questionnaires. The practice of the birth attendants during 371 deliveries was evaluated through direct observation by a researcher, using a standardized observational checklist with predefined criteria. Data were analyzed using SPSS version 20, and chi-square and one-way analysis of Variance (ANOVA) tests were applied, with statistical significance set at *P* ≤ 0.05.

**Results:**

The mean knowledge score of the participants was 15.04, and there was no significant statistical difference between the three groups birth attendants in this regard (*P* = 0.230). The mean attitude score was 69.00. 66% of the participants had a positive attitude towards the partograph, and a significant statistical difference was observed between birth attendants (*P* = 0.001). Also, 57.2% of the partograph forms were not completed during labor, 39.6% were incomplete, and only 3.2% of the forms were completed fully and standardly. The chi-square test showed that there was a significant statistical relationship between the type of birth attendance and the completion of the partograph (*p* = 0.001). The duration of the first stage of labor, the 1st-minute Apgar score, postpartum hemorrhage, the need for resuscitation, and the need for Neonatal Intensive Care Unit (NICU) admission were significantly associated with the completion of the partograph (*P* < 0.05).

**Conclusions:**

According to the results of this study, despite the acceptable knowledge and attitude scores of obstetric caregivers, the level of partograph use was still very low. Alongside continuous training, appropriate policy-making and structured implementation protocols are essential to ensure optimal partograph use and improve maternal and neonatal outcomes.

**Supplementary Information:**

The online version contains supplementary material available at https://doi.org/10.1186/s12884-026-09569-1.

## Background

Reducing maternal and neonatal mortality has long been emphasized as an indicator of the quality of prenatal care, and achieving this remains a key target of the Sustainable Development Goals (SDG 3.1 and 3.2) [1]. The major causes of maternal mortality are largely preventable through evidence-based interventions. The five leading direct causes of maternal death are hemorrhage, hypertension, sepsis, abortion, and obstructed or prolonged labor [2]. Prolonged labor, with or without uterine rupture, ranks among the primary causes of maternal death in most low- and middle-income countries, although its prevalence varies across regions [3]. In this regard, the World Health Organization (WHO) has made strong recommendations, including developing the concept of the partograph and emphasizing the need for its use [4]. Partograph is a simple and low-cost monitoring tool for intrapartum care recommended by the WHO with the potential to identify complications of labor by graphically displaying vital maternal issues and labor progress. Also, the routine use of the partograph provides better decision-making for the diagnosis and management of labor [5]. The use of this tool in labor and childbirth has a history of more than 4 decades, but if not used correctly, it can have a negative impact on the final outcomes [6].

In 2020, WHO introduced the Labor Care Guide as a more updated approach to monitoring labor, with a greater focus on individualized and evidence-based care [7]. Nevertheless, the partograph is still routinely used in many healthcare settings, particularly in low- and middle-income countries, and continues to play an important role in monitoring labor progress. Despite its long-standing recommendation by WHO, studies have shown that the use of the partograph remains limited and often incomplete in many developing and less developed countries [4, 5, 8]. This evidence is mainly based on studies conducted in Africa and some countries in South and Southeast Asia [9], but reports on the status of partograph use in Southwest Asian countries are limited. Assessing the practical application of the partograph and its determinants is very valuable in designing appropriate intervention strategies to improve the quality of maternal care [8]. The effective use of any tool, including the partograph, is directly influenced by healthcare providers’ knowledge and attitudes towards it. Assessing the current status of partograph utilization by birth attendants, particularly in resource-limited settings like Iran, is crucial for effectively reducing maternal and neonatal complications during childbirth.

## Materials and methods

This cross-sectional (descriptive-analytical) study was conducted in winter 2023 to investigate the status of partograph utilization by birth attendants and their knowledge and attitudes towards it. The participants in this study were midwives, obstetrics residents, and midwifery students in the labor wards of four teaching hospitals affiliated with Ahvaz Jundishapur University of Medical Sciences in southwestern Iran. After ethical approval (IR.AJUMS.REC.1402.257) was received and the participants were briefed on the study objectives, all birth attendants working in the labor wards of the four aforementioned hospitals who were willing to participate in the study and had at least one month of work experience were invited to participate. After providing written informed consent, participants completed the self-administered knowledge and attitude questionnaires. They were informed that their responses would remain confidential and would be used solely for research purposes. No identifying information, including participant names, was recorded on the questionnaires to ensure anonymity. In these hospitals, the modified version of partograph is available in the labor wards and is included in the delivery records. The study included 155 participants: 31 obstetrics residents (19 in their first and second years and 12 in their third and fourth years), 73 midwives (62 holding a bachelor’s degree and 11 holding a master’s degree), and 51 final-year midwifery students. The same individuals who completed the questionnaires were also responsible for completing the partographs.

In this study, the knowledge questionnaire was adapted from the content of the partograph form. It consisted of 23 items, each scored as either correct (1) or incorrect (0). The total knowledge score ranged from 0 to 23 and was categorized into: poor (0–7), moderate (8–15), and good (16–23). Open-ended questions were included to inquire about previous training related to the partograph form, perceived need for further training, and participants’ self-reported practice in completing the form.

The attitude questionnaire used in this study contained 18 questions scored based on a 5-point Likert scale, with a total score ranging from 18 to 90. The total attitude score ranged from 18 to 90 and was categorized into: poor (18–41), moderate (42–65), and good (66–90). Additional general questions were included to assess participants’ perceptions of training opportunities, the availability of the partograph form, their ability to interpret the partograph, and the time taken to complete it.

A checklist consisting of 11 items was used to assess the completeness of partograph documentation. The completion status was categorized into three levels: complete, when all 11 items were recorded according to WHO standards and completed during labor; incomplete, when at least one but not all items were recorded; and not completed, when no partograph record was documented. The checklist evaluated the recording of demographic information, cervical dilation, fetal head descent, fetal heart rate, warning line and actions, maternal vital signs, status of the amniotic sac, uterine contractions, oxytocin drops, and medications administered to the mother. The checklist also recorded the time of completion, accuracy of recording, and the individual who completed the partograph (midwife, resident, or midwifery student).

The knowledge and attitude questionnaires were developed based on a review of relevant literature [10, 11]. The reliability of the knowledge questionnaire was assessed using the Kuder–Richardson 20 (KR-20) coefficient and was found to be 0.878, while the internal consistency of the attitude questionnaire was evaluated using Cronbach’s alpha coefficient, which was 0.958. To improve the appropriateness and clarity of the instruments, both questionnaires were reviewed by 15 faculty members with expertise in midwifery and maternal health, and their feedback was considered in the final version. All completed questionnaires were reviewed for completeness at the time of collection. To assess the actual completion of partographs, direct observation was employed to minimize reliance on retrospective records and also researcher was not involved in patient care or clinical decision-making during the observations conducted in the four hospitals. The lead researcher was present during all three work shifts and observed eligible women with singleton pregnancies who had reached 4 cm cervical dilation at admission to the labor ward. Data collected included the completion of the partograph, duration of the first and second stages of labor, 1st- and 5th-minute Apgar scores, mode of delivery, perineal condition, need for labor induction, postpartum hemorrhage, and the need for neonatal intensive care unit (NICU) admission.

In this study, the sample size was determined to be 371 deliveries using the statistical software MedCalc, with a 95% confidence level and a 5% precision [8]. This sample size was proportionally allocated to the four mentioned hospitals, resulting in a range of 70 to 90 deliveries per hospital based on the previous month’s delivery numbers. Data were analyzed using SPSS version 20. Descriptive statistics were used to summarize the data, and inferential analyses were performed using chi-square tests for categorical variables and one-way ANOVA for continuous variables. The level of statistical significance was set at *P* ≤ 0.05.

$$\:\frac{{\left({z}_{1-\raisebox{1ex}{$\alpha\:$}\!\left/\:\!\raisebox{-1ex}{$2$}\right.}\right)}^{2}\times\:p\left(1-p\right)}{{e}^{2}}$$

$$\:{z}_{1-\raisebox{1ex}{$\alpha\:$}\!\left/\:\!\raisebox{-1ex}{$2$}\right.}=1.96$$

* p=0.43*

* e=0.05*

## Results

In the present study, the mean age of the participants (*N* = 155) was 29.2 ± 13.7 years. Regarding training, 68.5% of participants reported no formal training on partograph use, while 31.5% reported acquiring their knowledge from more experienced colleagues. Also, 75.9% stated that the partograph form was always available in delivery records. With regard to additional training, 48.1% of participants felt the need for additional training, and 57.4% believed they could interpret all parts of the partograph. Only 26.5% of participants always used the partograph during labor, and 43.2% completed the form after delivery. As far as knowledge was concerned, 9.9% of participants had poor knowledge, 32.7% had moderate knowledge, and 57.4% had good knowledge of the partograph. With respect to attitude, 6.2% of participants had a poor attitude, 27.8% had a moderate attitude, and 66% had a good attitude towards completing the partograph.

Among the 371 observed deliveries, no partograph was completed in 210 (56.6%) cases, 149 (40.2%) partographs were incomplete, and only 12 (3.2%) were completed fully and according to standard recommendations. For incomplete partographs, birth attendants typically only recorded dilation, fetal head descent, and fetal heart rate. Also, 89.9% of partographs were completed immediately after delivery, while only 10.1% were completed during labor ( Table 1).

**Table 1:** Completion status of partograph components among incomplete partographs in Ahvaz teaching hospitals, 2023 (*N* = 149)

Checklist Items	Complete *n* (%)	Incomplete *n* (%)	Not recorded *n* (%)
Recording of demographic information	10 (6.7%)	11 (7.4%)	128 (85.9%)
Recording of cervical dilation	15 (10.1%)	134 (89.9%)	-
Recording of fetal head descent	11 (7.4%)	138 (92.6%)	-
Recording of fetal heart rate	14 (7.4%)	135 (90.6%)	-
Attention to warning line	-	-	149 (100%)
Recording of maternal vital signs	-	-	149 (100%)
Recording of status of membranes	-	-	149 (100%)
Recording of uterine contractions	-	-	149 (100%)
Recording of medication received	-	-	149 (100%)
Recording of maternal medications	-	-	149 (100%)
Time of partograph completion	15 (10.1%)	134 (89.9%)	-

The study included 161 deliveries by obstetrics residents, 124 by midwives, and 86 by midwifery students. The average maternal age was 26.9 ± 6.5 years, with an average of 2.58 ± 1.46 pregnancies. The mean gestational age was 38.14 ± 1.13 weeks, and the average birth weight was 3332 ± 369.72 g.

The study found significant gaps in knowledge and training regarding the partograph among birth attendants. The partograph was not consistently used or completed correctly, with most being completed after delivery rather than during labor.

As shown in Table 2, there was no significant difference in the mean scores of the knowledge questionnaire among the different groups of birth attendants (*P* = 0.230). However, there was a significant difference in terms of attitude (*P* < 0.001). The lowest mean knowledge and attitude scores were for midwives, while the highest scores were for midwifery students. Residents had the weakest practice in completing the form, and there was a significant difference in how the form was completed among the different groups (*P* < 0.001).

**Table 2:** Knowledge, attitude, and partograph completion practice among birth attendants in Ahvaz teaching hospitals, 2023 (*N* = 155)

Group	Knowledge (Mean ± SD)	Attitude (Mean ± SD)	Practice		
			Complete	Incomplete	Not recorded
Resident	15.19(4.52)	65.16(16.43)	0	4	157
Midwife	14.34(5.65)	64.07(17.1)	9	111	7
Midwifery Student	15.35 ± 5.33	76.34 ± 10.60	3	34	46
Statistical Test*	*P* = 0.230*	*P* = 0.001*	*P* = 0.001**		

*Analysis of Variance Test

**Chi-square Test

According to Table 3, this study found that based on one-way analysis of variance, the duration of the first stage of labor and the 1st-minute Apgar score were significantly associated with the completion of the partograph (*P* < 0.05).

**Table 3:** Association between partograph completion status and maternal and neonatal outcomes among observed deliveries in Ahvaz teaching hospitals, 2023 (*n* = 371)

Variable	Not recorded	Incomplete recorted	Complete recorded	Pvalue*
Partograph Use	210(%56.6)	149(%40.2)	12(%3.2)	
1st Stage Labor Duration (hours)	3.89	3.40	2.91	0.001*
2nd Stage Labor Duration (minutes)	37.24	38.87	31.25	0.265*
Birth Weight (grams)	3324	3348	3262	0.666*
Apgar Score at 1 min	8.4	8.99	9	0.01*
Apgar Score at 5 min	9.3	9	10	0.175*
Need for Resuscitation	Yes = 15 No = 202	Yes = 1 No = 151	Yes = 0 No = 2	0.01**
Need for NICU Admission	Yes = 15 No = 202	Yes = 1 No = 151	Yes = 0 No = 2	0.01**
Postpartum Bleeding	L*** 23(10.7%)	L *** 9(6.0%)	L *** 0(0.0%)	0.004**
	M ****188(88.4%)	M **** 130(89.3%)	M ****10(83.3%)	
	S***** 2(0.9%)	S***** 7(4.7%)	S***** 2(16.7%)	

*Result of one-way analysis of variance

**Chi-square Test

***Light: The amount of blood and lochia is from 10 to 25 cc or less than 10 centimeters of blood-soaked pad

****Moderate: The amount of blood in the vagina is 25 to 50 cc or less than 15 cm from the blood-soaked pad

*****Sever: A pad becomes completely soaked with blood within 15 min, or blood has pooled under the mother’s buttocks [12]

In addition, the results of the chi-square test showed that postpartum hemorrhage, the need for resuscitation, and NICU admission were significantly associated with partograph completion (*P* < 0.05) (Table 3). It is important to emphasize that these findings reflect associations only. Given that the majority of partographs were completed after delivery and that potential confounding factors (e.g., maternal health status, staffing levels, and workload) were not controlled for, these associations should not be interpreted as causal.

## Discussion

The aim of this study was to identify the status of knowledge, attitude, and practice of birth attendants regarding the use of partographs as a tool to empower healthcare providers in monitoring the progress of labor, performing timely interventions, and making decisions to refer mothers. Despite exhibiting relatively good knowledge (57.4%) and attitude (66%), birth attendants in this study demonstrated poor practice in completing the partograph form. Only 3.2% fully completed the form, while it was left entirely incomplete in 56.6% of cases.

Despite the WHO’s emphasis on partograph use, particularly in low and middle-income countries, numerous studies have demonstrated low rates of both utilization (around 31%) and correct completion (approximately 3%) [5]. Although half of the participants in this study were obstetric residents and midwifery students who obtained a good overall average score in knowledge about the partograph, the majority of birth attendants stated that they had not received formal training on the partograph and had acquired information in this area in the workplace and from more experienced colleagues. In line with these findings, a study in Nigeria also showed that although 70.8% of the participants had good knowledge of the partograph, the majority of them could not locate the action line and alert line [13]. The results of another study showed that individuals who received in-service or on-the-job training were 5.5 times more likely to have a good level of knowledge about the partograph compared with those who did not receive training [14], which is consistent WHO recommendations highlighting the importance of regular and continuous training for birth attendants [15]. The results of this study indicated that having good knowledge and attitude towards the partograph does not guarantee its correct use. Importantly, the discrepancy observed between adequate knowledge and poor practice suggests that barriers to partograph use are not limited to individual-level factors. In the present study, most forms were completed after delivery, indicating that real-time documentation was not routinely performed. This pattern strongly suggests the influence of systemic constraints rather than knowledge deficits alone. High workload, staff shortages, time pressure during labor management, and duplication of documentation have been identified as major barriers to effective partograph use in similar settings [16, 17].

According to our results, although midwives had the lowest average scores in knowledge and attitude towards the partograph, they performed better than the other two groups (i.e., residents and midwifery students) in completing the partograph. Although no study was found that compared knowledge, attitude, and practice towards the partograph among different groups of birth attendants, one study showed that, in general, physicians and midwives have a positive attitude towards it [18]. Certainly, the difference in using the tool could be influenced by the conflicting roles and responsibilities of birth attendants as midwives, residents, or midwifery students in managing labor and delivery, but in general, there are conflicting results in this area. For example, in a study by Ako et al. (2022), despite their positive attitude, the practice of birth attendants in completing the partograph was reported to be poor [19]. However, Ayehubizu et al. (2022) reported that the negative attitude of the majority of birth attendants was associated with their poor practice in completing the form, and those who had a positive attitude were three times more likely to use the partograph than others [10]. This may reflect differences in professional roles and experiential learning. Midwives are more consistently involved in continuous labor monitoring, leading to greater procedural familiarity and routinization of partograph completion. In contrast, residents and midwifery students, despite higher theoretical knowledge, have more intermittent exposure to labor documentation due to their training structure and rotating responsibilities. This highlights that partograph utilization is more strongly influenced by role allocation and clinical exposure than by knowledge or attitude alone [15, 16, 20].

In the present study, a total of 40% of the forms had at least one component of the partograph completed, and in most incomplete forms, FHR and cervical dilatation were recorded more than other components of the partograph. A study in Ethiopia reported the use of the partograph at 69%. In this study, a completed partograph was defined as a form in which at least one recording of FHR, cervical dilatation, uterine contractions, vital signs, amniotic fluid status, and fetal head descent [8]. In contrast, a study in India reported a much lower rate of partograph completion, with 6% of the recorded forms having at least one assessment of cervical dilatation [21]. It has been reported that in the review of incomplete partographs, FHR, cervical dilatation, and uterine contractions were recorded more than other components of the partograph [22]. Overall, these findings suggest that the partograph is often used in a selective rather than complete manner, with healthcare providers focusing mainly on a few key clinical indicators instead of documenting all required components.

Although out of the 371 deliveries examined, 210 had incomplete partograph records and only 12 were fully completed according to standards, some differences in birth outcomes were observed according to the level of completion. Fully completed partographs were associated with a shorter duration of the first stage of labor and higher 1-minute Apgar scores. In addition, better outcomes were observed in terms of reduced need for neonatal resuscitation, NICU admission, and postpartum hemorrhage. These findings are in line with previous studies showing that appropriate use of the partograph is associated with improved neonatal outcomes and a reduction in adverse events such as neonatal asphyxia and prolonged labor [9]. However, evidence on its relationship with specific stages of labor remains limited and inconsistent across studies.

The low rate of complete utilization observed in this study may reflect challenges in integrating the partograph into routine clinical practice in similar settings. When used properly, the partograph is intended to support timely decision-making and improve the quality of intrapartum care, which can ultimately contribute to better maternal and neonatal outcomes [23, 24].

Strengths and Weaknesses of the Study:

This study was conducted in teaching hospitals where, in addition to midwives, obstetric residents and midwifery students were involved in the documentation of the birth process. Differences in roles, decision-making authority, and accountability among these groups may have influenced their commitment to using the partograph.

One of the strengths of this study was the use of direct observation and real-time assessment of partograph completion among 371 deliveries. Unlike many previous studies that relied on retrospective data or medical record reviews, this approach allowed a more objective evaluation of actual practice. However, the presence of the researcher during observations may have influenced the behavior of birth attendants and their completion of the partograph.

Several potential confounding factors, including maternal characteristics and birth-related factors, were collected and evaluated. However, due to the cross-sectional design of the study and the limited number of fully completed partographs (*n* = 12), multivariable analysis could not be performed to adjust for all possible confounding factors. Therefore, the observed associations between partograph completion and maternal and neonatal outcomes should be interpreted with caution and further studies with larger sample sizes are needed to confirm these findings.

## Conclusion

According to the findings of this study, although birth attendants had relatively good knowledge and positive attitudes toward the partograph, its complete and standard use during labor was limited, showing a gap between knowledge and actual practice. These findings suggest that training alone may not be sufficient, and continuous support, regular supervision, and clear clinical protocols are needed to improve the routine use of the partograph. Strengthening these measures may contribute to better quality of intrapartum care and improved maternal and neonatal outcomes.

## Supplementary Information

Supplementary Material 1.

## Data Availability

No datasets were generated or analysed during the current study.
