# Supplementary material for: Knowledge, attitude, and practice of birth attendants regarding partograph use and its association with birth outcomes: a cross sectional study in Iran
Source: BMC Pregnancy Childbirth. 2026 Jul 3;26:1069. doi: 10.1186/s12884-026-09569-1 (PMC13617867; doi:10.1186/s12884-026-09569-1)
Supplement: Supplementary file 1 [file 12884_2026_9569_MOESM1_ESM.docx]

The questionnaires were developed based on a review of relevant literature.

Knowledge Questionnaire

In this study, the knowledge questionnaire was adapted from the content of the partograph form. It consisted of 23 questions, each with a correct or incorrect answer (scored 1 or 0, respectively). The total score ranged from 0 to 23. Open-ended questions were included to inquire about previous training related to the partograph form, perceived need for further training, and participants' self-reported performance in completing the form.

Knowledge

1-When should a partograph be fill?

a- At the time of admission to the labor ward

b- Latent phase

c- Active phase

d- After birth

2-Who is responsible for filling out the partograph?

a-Midwives	b- Midwifery students	c- Gynecology residents	d- Medical students

e-Anyone who is responsible for the monitoring of labor.

-Which of the following can be diagnosed by filling out a partograph?

3- prolonged labor	a- Yes	b- No	c- Don't know

4- Arrested labor	a- Yes	b- No	c- Don't know

5- Labor dystocia	a- Yes	b- No	c- Don't know

6- Insufficient uterine contractions	a- Yes	b- No	c- Don't know

7-Fetal distress	a- Yes	b- No	c- Don't know

8- Abnormal fetal heart rate	a- Yes	b- No	c- Don't know

9- Satisfactory progress of labor	a- Yes	b- No	c- Don't know

10- Need for augmentation	a- Yes	b- No	c- Don't know

11- Cesarean section	a- Yes	b- No	c- Don't know

12-Maternal intake	a- Yes	b- No	c- Don't know

13- Characteristics of a partograph with normal labor………….

a- The progress line should be placed to the left of the alert line.

b- The progress line should be placed on the alert line.

c-The progress line should be placed to the right of the alert line.

- What should be the characteristics of a normal labor in the partograph ?

14-Three Three to five contractions every 10 minutes is normal	a- Yes	b- No	c- Don't know

15-The duration of a contraction ok is 40-60 seconds	a- Yes	b- No	c- Don't know

16- We need 10 minutes to assess the adequacy of uterine contractions. a- Yes	b- No	c- Don't know

17-Progress of labor is assessed by dilation and descent	a- Yes	b- No	c- Don't know

18- arrested Labor if dilation does not change for 4 hours	a- Yes	b- No	c- Don't know

19- In stage two, the fetal heart rate is recorded every half hour	a- Yes	b- No	c- Don't know

20-Amniotic fluid check at each examination	a- Yes	b- No	c- Don't know

21-Dilation is recorded every 2-4 hours	a- Yes	b- No	c- Don't know

22- Fetal head descent is recorded every 2 -4 hours	a- Yes	b- No	c- Don't know

23-Check maternal vital signs every 4-hour	a- Yes	b- No	c- Don't know

Open-ended questions

1- Have you received training on how to fill out the partograph? a- Yes	b- No

2-If your answer is yes, who did you receive training from?

a- Experienced colleagues	b- Course at university	c- Training by midwifery professors in the clinic	d- Books, educational resources, internet	e- Training by gynecologists

3- Do you need an additional training on how to use the partograph? a- Yes	b- No

4-Do you use a partograph to monitor women in labor? a- Always	b- Often	c- Sometimes

d- Rarely	e- Never

Attitude Questionnaire

The attitude questionnaire used in this study contained 18 questions scored based on a 5-point Likert scale, with a total score ranging from 18 to 90. To better understand the levels of knowledge and attitude regarding the partograph, the score ranges were divided into three categories: poor, moderate, and good. Additional general questions were included to assess participants' perceptions of training opportunities, the availability of the partograph form, their ability to interpret the partograph, and the time taken to complete it.

Attitude

To what extent do you agree with the following statements?

| Strongly disagree  1 | Disagree  2 | Don't know  3 | Agree  4 | Strongly agree  5 | Items |
| --- | --- | --- | --- | --- | --- |
| 1 | 2 | 3 | 4 | 5 | 1-Partograph should be used routinely in the labor ward. |
| 1 | 2 | 3 | 4 | 5 | 2- The use of partograph in the labor ward is of great importance. |
| 1 | 2 | 3 | 4 | 5 | 3-The use of partograph is essential for decision-making in labor. |
| 1 | 2 | 3 | 4 | 5 | 4-A partograph is a tool for monitoring the progress of labor. |
| 1 | 2 | 3 | 4 | 5 | 5-A partograph is a tool for diagnosing complications during labor. |
| 1 | 2 | 3 | 4 | 5 | 6-Partograph improves labor management. |
| 5 | 4 | 3 | 2 | 1 | 7-filling the partograph in the labor ward is not practical. |
| 1 | 2 | 3 | 4 | 5 | 8-The partograph is a desirable tool because it alerts the gynecologist/midwife to any deviations from normality. |
| 1 | 2 | 3 | 4 | 5 | 9-Midwives/gynecologists are able to identify problems during labor and diagnose early complications using partographs. |
| 1 | 2 | 3 | 4 | 5 | 10-Filling out the partograph by the midwife/gynecologist should be part of the rules of the labor ward. |
| 1 | 2 | 3 | 4 | 5 | 11-The use of partographs enables midwives to perform necessary interventions and to know when to refer to higher levels. |
| 5 | 4 | 3 | 2 | 1 | 12-Using a partograph is not beneficial because the estimate it provides is overwhelming. |
| 1 | 2 | 3 | 4 | 5 | 13-Filling the partograph during labor reduces maternal complication. |
| 1 | 2 | 3 | 4 | 5 | 14-Filling the partograph during labor reduces maternal mortality. |
| 1 | 2 | 3 | 4 | 5 | 15-filling a partograph during labor reduces neonatal complications. |
| 1 | 2 | 3 | 4 | 5 | 16-filling a partograph during labor reduces neonatal death. |
| 1 | 2 | 3 | 4 | 5 | 17-The partograph is a tool to improve the quality of care for women in labor. |
| 1 | 2 | 3 | 4 | 5 | 18-Partograph is one of the tools of the Safe Motherhood Program. |

Open-ended questions

1- Have you received any training on how to use a partograph during your work or education?

a- Yes	b- No

2-Is the partograph always available in delivery records? a- Yes	b- No

3- Are you able to interpret all parts of a partograph? a- Yes	b- No

4- When do you fill out the partograph?

a- After delivery	b- At the end of the shift	c- Immediately after any examination or intervention d- Whenever I have the opportunity

Observation checklist

| Checklist Items | Complete | Incomplete | Not Recorded ( |
| --- | --- | --- | --- |
| 1-Recording of demographic information |  |  |  |
| 2-Recording of cervical dilation |  |  |  |
| 3-Recording of fetal head descent |  |  |  |
| 4-Recording of fetal heart rate |  |  |  |
| 5-Attention to warning line |  |  |  |
| 6-Recording of maternal vital signs |  |  |  |
| 7-Recording of status of membranes |  |  |  |
| 8-Recording of uterine contractions |  |  |  |
| 9-Recording of medication received |  |  |  |
| 10-Recording of maternal medications |  |  |  |
| 11-Time of partograph completion |  |  |  |
